# Single-cell immune atlas for human aging and frailty

**DOI:** 10.1093/lifemedi/lnac013

**Published:** 2022-06-28

**Authors:** Sean X Leng, Graham Pawelec

**Affiliations:** Johns Hopkins Center on Aging and Immune Remodeling and Division of Geriatric Medicine and Gerontology, Department of Medicine, Johns Hopkins University School of Medicine, Baltimore, MD 21224, United States; Department of Immunology, University of Tübingen, Tübingen 72076, Germany; Health Sciences North Research Institute, Sudbury, ON P3E 2H2, Canada

Aging is associated with significant immune functional decline and remodeling, largely driven by alterations of gene expression and regulation of immune cells. For decades, researchers have investigated the dynamics of gene expression of immune cells through aging, both in humans and in mice. However, most of these studies were done at a bulk-cell level, which were influenced by the proportions of altered immune cell subsets. In addition, only limited inferences can be drawn from observations in mice because the genomic and environment background of laboratory mice are artifactually limited while human beings have diverse genetic background as well as individualized living environments, nutrition, gut microbiota, latent infections, socioeconomic circumstances, and many other lifestyle factors [1]. With the recent advent of single-cell multi-omics technologies, analyses of gene expression of individual immune cells have been performed in great detail at high resolution [2], [3]. For example, Liu and his colleagues studied age-related alternations in gene expression in multiple tissues and organs at the single-cell level and have constructed an Aging Atlas database as a resource [4].

**Figure 1.**
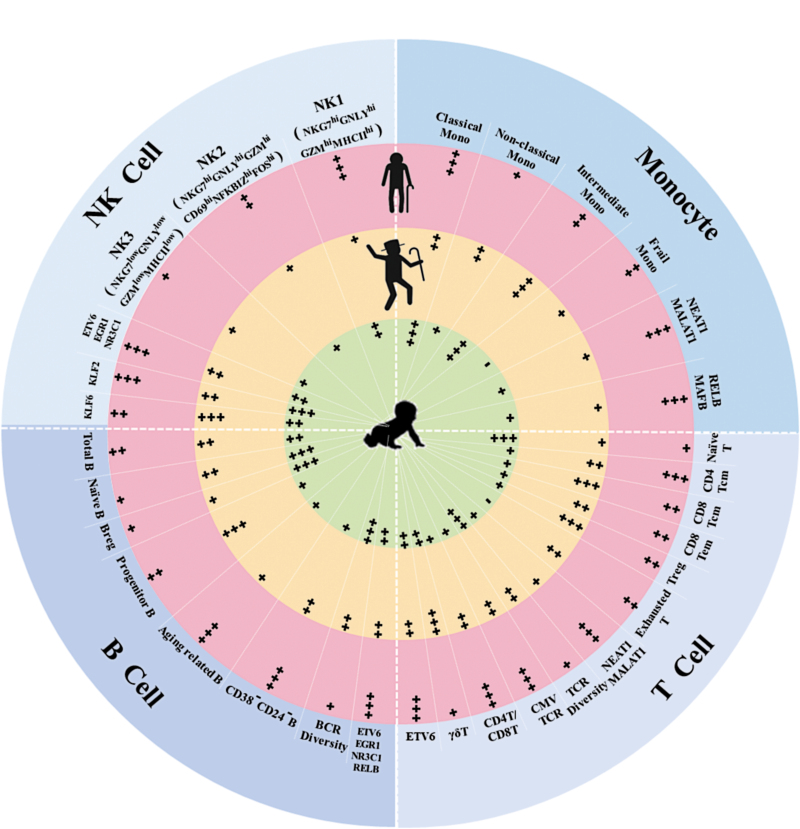
Gene expression signatures and functional characteristics of immune cells in neonates (cord blood, light green), healthy aging (light orange), and frailty (pink).

By convention, chronological age is used as the indicator of aging, as do most prior studies of the relationship between aging and immunity. However, everyone ages differently and chronological age does not address the tremendous heterogeneity of the older adult population. Frailty is a common geriatric syndrome and poor health status characterized by decreased physiologic reserve and increased vulnerability, leading to adverse health outcomes [5]. Biologically, frailty serves as a useful tool to address the heterogeneity of aging and associated changes and maladaptation of multiple systems, including the immune system, beyond the chronological age. For example substantial evidence suggests that chronic low-grade inflammatory phenotype (CLIP) plays an important role in the pathogenesis of frailty [6]. Other immune aging parameters have also been linked to poor health status, such as frailty (reviewed in Chen et al.) [7]. In the latest study recently published in Nature Aging, Luo and colleagues performed a comprehensive single-cell transcriptomic and TCR repertoire analysis, identifying gene expression signatures and functional characteristics of immune cell subsets along the whole spectrum of the aging process, from neonates (using cord blood) and young adults as controls to two groups of older adults who either appeared to be healthy (“healthy aging”) or who were frail (“frailty”) with similar chronological age (85.8 ± 11.1 years vs 88 ± 5.8 years, respectively) (Fig. 1) [8].

Similar to the study of Zheng [9], Luo et al. confirmed previous findings of different distributions of immune cell subsets at different ages, such as decreased proportions of naïve cells, and increase of memory and inflammatory cells. However, it was difficult to distinguish between the healthy and frail older adult groups in terms of immune cell subset population size, except for slightly increased CD4+ Tcm frequency in the frail group. As expected, an age-dependent accumulation of cellular heterogeneity and transcriptomic variability across the immune cell subtypes was evident. Compared to the healthy older adult group, a set of frailty-specific gene expression differences in 16 immune cell subsets was identified. These results, for the first time, demonstrate significant differences in gene expression dynamics of major immune cells between healthy aging and frailty. The authors also identified characteristic transcription factors (TFs) in various immune cell subtypes of certain age groups based on the specific expression of gene sets. Furthermore, transcriptional regulatory network analysis showed increased expression of TFs of the AP-1 and NF-κB families by immune cells in the frail older adult group, adding molecular evidence supporting a potential role of immune and NF-κB pathway activation in contributing to CLIP in frailty.

**Figure 2.**
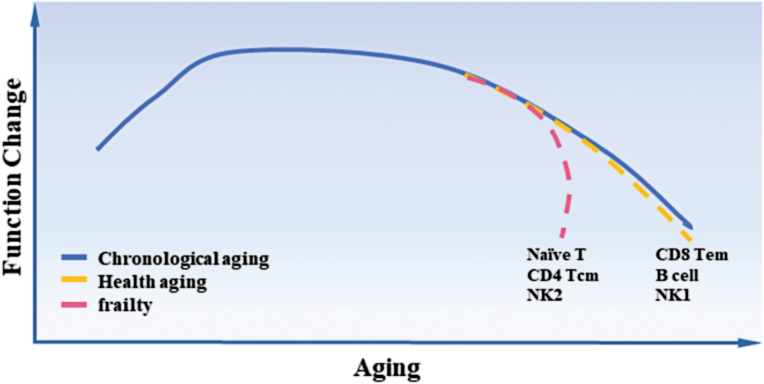
Different pace of immune cell aging and frailty.

Similar to the heterogeneity and variability of gene expression through aging, the immune cell subsets from healthy aging and frailty were of distinct aging dynamics and fates. Based on the results from the cell fate trajectory analysis (“pseudotime analysis”), the aging process of immune cells showed two major modes. In one mode, the pace of gene expression changes matches with the chronological age, such as for B, CD8 Tem, and NK1 cells. The other mode indicates dramatic gene expression alterations at a specific point in time, such as for naïve T, CD4 Tcm, and NK2 cells (Fig. 2). More in-depth studies are needed to further explore how the gene expression characteristics of each immune cell subset contribute to the distinct aging trajectory, and clarify whether the differential pace results in distinct functional outcomes.

Among the immune cells, T lymphocytes are affected broadly by aging. Frequencies of certain T-cell subsets altered significantly during aging, including the well-documented decrease of naïve CD8 T cell and increase of CD4 Tcm and exhausted T cells. CD4 Tcm and CD8 Tem frequencies also changed with frailty and aging. As such, the ratio of CD4 Tcm over CD8 Tem could potentially serve as a biomarker. Weather such change of CD4 Tcm/CD8 Tem ratio corresponds to the change of the well-documented CD4/CD8 ratio remains to be investigated. The observed decrease of TCR repertoire diversity from healthy aging to frailty could potentially serve as a biomarker for frailty as well. Another interesting finding from the study is the shared T-cell clones between healthy aging and frailty. It is not surprising that the same T-cell clones, which are defined by their possession of the exact same TCR sequences, were found in both naïve and memory T cell pools, as this is the basis of physiological adaptive immunity. However, the study also demonstrated significantly increased shared T-cell clones among different T-cell subsets, especially shared TCR between CD4+ and CD8+ T cells. Considering the dramatic decrease of TCR repertoire size from approximately 10^8^ in young adults to 10^6^ in frail older adults, the immune system may need a compensatory mechanism to meet constant pathogen challenges. The fact that T cells exhibit different gene expression profiles during aging and yet more additional ones in frailty, coupled with the fact that most of the shared TCRs were specific to cytomegalovirus (CMV), which is essentially ubiquitous in older adult populations in China, suggests significant pluripotency and resiliency of those T cells. This agrees with previous studies that observed cytotoxic function of CD4+ T cells in centenarians [10], suggesting that T cells in an immunosenescent state have the potential to be pluripotent.

Unlike T cells, B lymphocyte frequencies showed no significant difference between healthy aging and frailty. This is in contrast to previously reported decrease of naïve B and Breg cells and increase of aging related B and CD38-CD24-B cells in flow cytometry-based studies. Although gene expression analysis by Luo et al. showed some differences in B cells, effects of aging and frailty on B cells need further investigation. For NK cells, one subset identified as CD69^low^MHC-II^hi^ was more abundant in frail older adults, suggesting an exhausted status of NK cells associated with frailty.

Single-cell technology can help to discover rare cell subtypes or subtypes that have never been reported before. In this study, a subpopulation of frailty-specific monocytes was identified, which might be derived from the classical monocyte population. The gene expression pattern of this frailty-specific monocyte subset was different from any of the defined classical, intermediate, or non-classical monocytes, particularly with regard to high-level expression of the long non-coding RNA (lncRNA) NEAT1 and MALAT1, and transcription factors ZEB2, NFKB2 and REL, and their low-level expression of CST3, FTL and CCL4, and MHCII genes. However, no specific cell surface markers are known to identify this frailty-specific monocyte subset at the present time. Further studies are indicated to translate these intriguing finding to the protein and functional levels.

These highly expressed lncRNAs, NEAT1, and MALAT1 (otherwise known as NEAT2) in frailty-specific monocytes and exhausted T cells raise several interesting questions. Because their altered expression has also been observed in several types of tumor cells, it is important to understand the relationships between high NEAT1 expression in tumorigenesis and senescence. The question of why only monocytes and exhausted T cells express high levels of NEAT1 is also worth noting. Considering the role of NEAT1 in the formation of paraspeckle backbone in the nuclear interchromatin space, more studies are warranted to explore the potential role of paraspeckle in aging and cellular senescence.

In summary, the study of Luo et al. developed a comprehensive single-cell transcriptomic and TCR repertoire atlas of human immune cells from birth, young adults, and to older adults who are apparently healthy and those who are frail at a similar chronological age. It provides a basic resource for investigating the potential impact of the clinically defined syndrome of frailty on immune aging and vice versa. Additionally, considering the continuing challenge of COVID-19, which disproportionately impacts older adults with severe disease and mortality [7], such an atlas will be critically important in leveraging our understanding of immune aging for the design of improved interventions to optimize immune function in senior citizens.
